# Effects of Different Extraction Methods on the Molecular Composition and Biological Activities of Polysaccharides from *Pleione yunnanensis*

**DOI:** 10.3390/molecules30091942

**Published:** 2025-04-27

**Authors:** Zhiqin Song, Zhihong Zheng, Xue Han, Yaya Chen, Hai Liu, Lin Yang, Mingkai Wu

**Affiliations:** 1 Institute of Crop Germplasm Resources/Institute of Modern Chinese Herbal Medicines, Guizhou Academy of Agricultural Sciences, Guiyang 550006, China; szq2023@126.com (Z.S.); zzh_gzsn@163.com (Z.Z.); wingdezkl@163.com (X.H.); wmlove@163.com (Y.C.); lilylike0915@163.com (H.L.); 2 Guizhou Key Laboratory of Agricultural Biotechnology, Guizhou Academy of Agricultural Sciences, Guiyang 550025, China

**Keywords:** *Orchidaceae*, polysaccharides, extraction methods, antioxidant activity, hypolipidemic effect

## Abstract

In this study, polysaccharides from *Pleione yunnanensis* (*Orchidaceae*, abbreviated as *P. yunnanensis*) were obtained by five methods: hot water extraction, microwave extraction, cold water extraction, enzymatic extraction, and ultrasonic extraction. Their structural characteristics, antioxidant properties, and hypolipidemic activities were explored. The results showed that the five polysaccharides all exhibited typical infrared spectral characteristics of polysaccharides. Their monosaccharide compositions were basically the same, all consisting of glucose and mannose, but their surface morphologies differed significantly. The polysaccharide extracted by the enzymatic method had the lowest molecular weight but showed good antioxidant properties. The polysaccharides extracted by ultrasonic and cold water methods showed great potential for hypolipidemic effects. Different extraction methods had impacts on the physicochemical properties, biological activities, and microstructures of *P. yunnanensis* polysaccharides. In practical applications, it is necessary to select appropriate extraction methods according to different requirements. The results of this study provide a reference basis for the precise development and utilization of *P. yunnanensis* polysaccharides.

## 1. Introduction

*P. yunnanensis* belongs to the genus *Pleione* of the family *Orchidaceae*. It is a terrestrial or semi-epiphytic herb with high medicinal and horticultural values. Its pseudobulbs can be used medicinally and are commonly known as “Bingqiuzi”. It is mainly distributed in southwestern Sichuan, western to northern Guizhou, northwestern to southeastern Yunnan, and southeastern Tibet in China [1]. It was included under the item “Shancigu” in the 2020 edition of the Chinese Pharmacopoeia. Currently, researchers have isolated more than a hundred compounds of over ten types from *P. yunnanensis,* such as phenanthrenes, bibenzyls, glycosides, lignans, anthraquinones, flavonoids, and steroids [2,3,4]. Polysaccharides are natural components with diverse functions and good activities in *P. yunnanensis*. They have a relatively high molecular weight and exhibit significant biological activities in aspects such as antioxidant, immune regulation, antitumor, glycolipid metabolism, and intestinal flora regulation [5], and can be applied in various fields such as food and biomedicine [6]. It has been reported that the polysaccharide content in *P. yunnanensis* can be as high as over 50% [7]. In the field of plant research, exploring bioactive polysaccharide compounds has become a research hotspot. It has been reported that Shancigu polysaccharides can be used in the adjuvant treatment of liver cancer and other tumors, and as an excipient for traditional Chinese medicine compound preparations, for the adjuvant treatment of immunodeficiency or immune-compromised diseases [8,9].

The extraction process of plant polysaccharides is a crucial link in the research and development of polysaccharides. The activity of polysaccharides is closely related to their structure, and the extraction conditions affect the composition and microstructure of polysaccharides. Currently, the extraction methods for plant polysaccharides include water extraction, alkaline extraction, microwave-assisted extraction, ultrasonic-assisted extraction, etc. The existing studies have shown that different extraction methods have significant effects on the monosaccharide composition, molecular weight distribution, and glycosidic bond linkage patterns of polysaccharides from plants such as *mulberries*, *Bletilla striata*, and *Ziziphus jujuba* [10,11,12]. However, there are no reports on the comparative study of the structural characteristics and biological activities of *P. yunnanensis* polysaccharides.

Based on this, this study took the pseudobulbs of *P. yunnanensis* as the research object. Five *P. yunnanensis* polysaccharides (abbreviated as PYp) were obtained by microwave extraction, cellulase extraction, hot water extraction, ultrasonic extraction, and cold water extraction. Their chemical compositions, structural differences, and antioxidant and hypolipidemic activities were comparatively analyzed, aiming to provide a scientific basis for the further development and utilization of *P. yunnanensis* polysaccharides.

## 2. Results and Discussion

### 2.1. Protein, Total Polysaccharide, and Uronic Acid Contents

**Figure 1:**
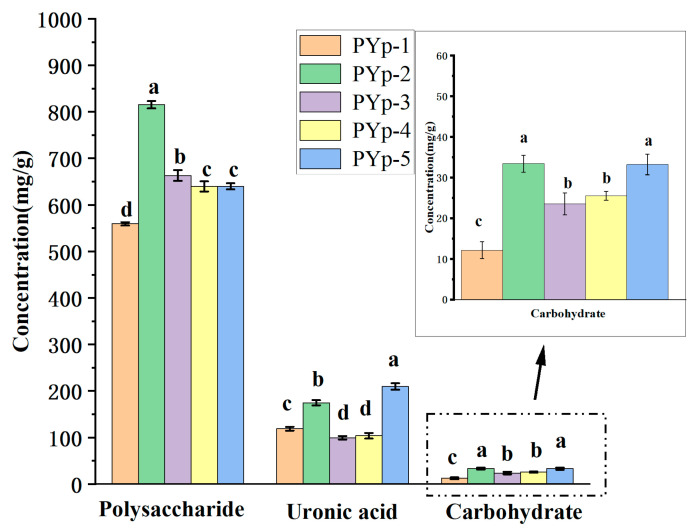
Comparative analysis of protein, total polysaccharide, and uronic acid contents in extracts obtained by different extraction methods. Different letters assigned to each bar indicate significant differences among groups (*p* < 0.05).

As shown in Figure 1, the chemical composition of the polysaccharides (PYp-1 to PYp-5) extracted from *P. yunnanensis* using different methods was analyzed. The total polysaccharide content varied significantly among the samples: PYp-1 exhibited the lowest content (55.94%), while PYp-2 showed the highest value, exceeding PYp-1 by more than 45%. This disparity may be attributed to the introduction of small-molecule impurities and other components during microwave-assisted extraction, which could increase compositional complexity and complicate the subsequent purification processes.

A uronic acid content analysis revealed that PYp-5 contained nearly twice the uronic acid levels of PYp-1, PYp-3, and PYp-4. Protein content was highest in PYp-2 (33.41 mg/g) and PYp-5 (33.22 mg/g), both exceeding 2.5 times that of PYp-1 (12.15 mg/g). Overall, PYp-2 demonstrated the highest values in total polysaccharide, protein, and uronic acid contents.

The contents of total polysaccharides and uronic acids obtained by the cellulase extraction method are relatively high. This enhancement may result from the enzymatic degradation of cellulose and pectin in the plant cell wall, facilitating a more efficient release of polysaccharides [13].

### 2.2. Molecular Weight and Monosaccharide Composition

**Figure 2:**
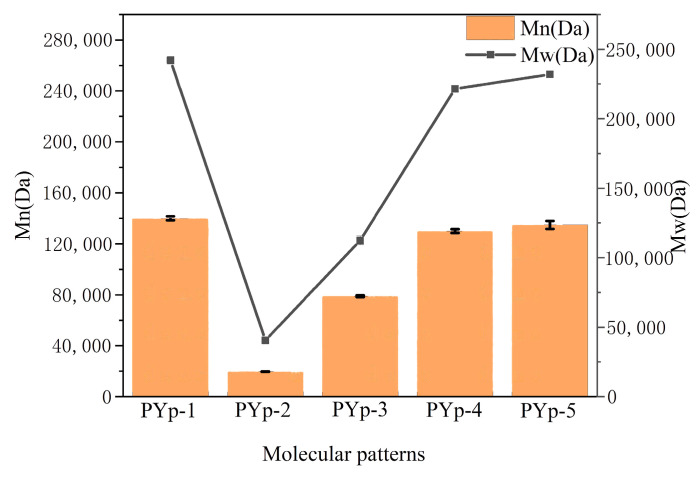
Molecular patterns of *P. yunnanensis* polysaccharides from five extraction methods.

The molecular weights of the PYps extracted by the five methods are shown in Figure 2. From the molecular weight detection results, it can be seen that the molecular weight range was 4.044 × 10^4^–2.443 × 10^5^. PYp-1 had the highest weight average molecular weight, and PYp-2 had the lowest. The molecular weights of the polysaccharides obtained by the other three extraction methods were all above 200 kDa. During the enzyme extraction process of PYp-2, glycosidic bonds were usually broken, which subsequently reduced the molecular weight. Therefore, PYp-2 had the lowest molecular weight. The polydispersity index (Mw/Mn) is an indicator for characterizing the width of the polymer molecular weight distribution and is positively correlated with the molecular weight distribution [12]. The PDI values of the five polysaccharides were 1.728, 2.064, 1.425, 1.704, and 1.720, respectively, which indicates that different extraction methods have an impact on the dispersity of the PYp molecular weight. The cellulase extraction method exhibited the largest dispersity index. In contrast to cellulase extraction, the other methods might have damaged the polysaccharide chains to varying extents during the extraction process. For instance, the high temperatures and long extraction times in those methods could cause such damage, resulting in a more uniform molecular weight distribution [14].

The components of Fucose (Fuc), Rhamnose (Rha), Arabinose (Ara), Galactose (Gal), glucose (Glc), Xylose (Xyl), mannose (Man), Fructose (Fru), Ribose (Rib), galacturonic acid (Gal-UA), glucuronic acid (Glc-UA), mannuronic acid (Man-UA), and guluronic acid (Gul-UA) in the five polysaccharides were analyzed, and the results are shown in Table 1.

The analysis of the monosaccharide composition showed that the five polysaccharides had the same types of monosaccharide composition, which was similar to the monosaccharide composition of reported *Orchidaceae* plants such as *Cremastra appendiculata*, *Bletilla striata*, and *Dendrobium officinale* [15]. They were mainly composed of glucose and mannose, and no other monosaccharides were detected in the results. However, there were slight differences in the molar ratios of the monosaccharides. The proportions of glucose and mannose in PYp-2 were similar, being 50.37% and 49.63%, respectively. The other four were mainly composed of mannose, with the proportion exceeding 54%, while the proportion of glucose was between 43 and 46%. The monosaccharide contents of PYp were 880.67, 847.22, 815.08, 854.18, and 834.35 μg/mg, respectively. However, no galacturonic acid, glucuronic acid, mannuronic acid, or guluronic acid were found. Combining with the results of the chemical composition analysis of monosaccharides, it can be known that PYp contains uronic acid, indicating that the uronic acid in PYp is of other types except for galacturonic acid, glucuronic acid, mannuronic acid, and guluronic acid.

Among the five extraction methods, PYp-2 was an enzymatic extraction method, while the remaining four were physical extraction methods. The results indicated that the enzymatic extraction method yielded higher Glc content, whereas physical extraction methods resulted in higher Man content. This finding aligns with previous studies on *Dendrobium officinale* and other *Orchidaceae* species, suggesting that different extraction methods have minimal effects on monosaccharide types but influence the monosaccharide composition of heteropolysaccharides [16].

### 2.3. Infrared Spectroscopy Analysis

**Figure 3:**
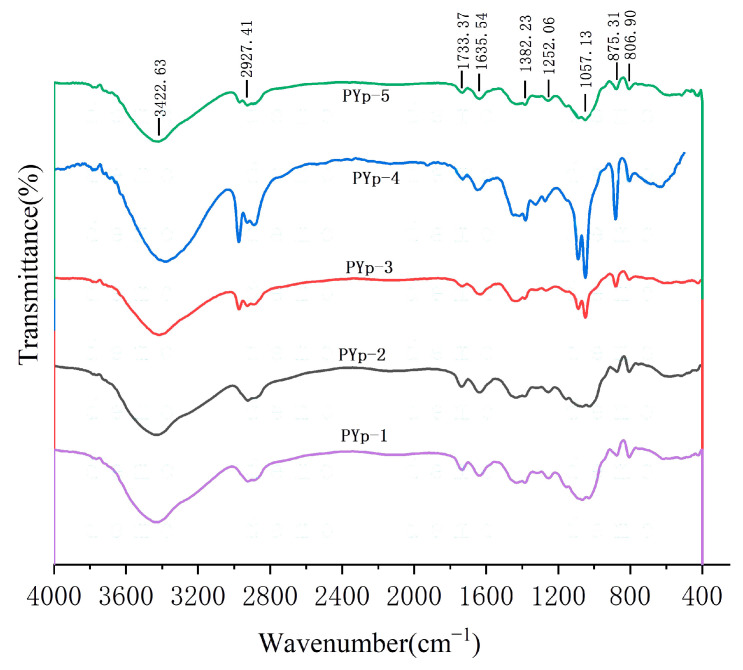
Infrared spectra of polysaccharides from *P. yunnanensis* obtained by different extraction methods.

The polysaccharides from *P. yunnanensis* extracted by the five methods all had similar characteristic absorption peaks of carbohydrate compounds, indicating that the main products obtained by the different extraction methods were all polysaccharides from *P. yunnanensis*. As shown in the figure, the relatively wide absorption peak formed around 3422 cm^−1^ was generated by the stretching vibration of O-H. The absorption peak at 2927 cm^−1^ was the C-H stretching vibration peak of methyl and methylene groups, which was a characteristic absorption peak of polysaccharides. The absorption peak around 1635 cm^−1^ was caused by the angular vibration of N-H in primary and secondary amines or the stretching vibration of C=O in carboxyl groups. From this, it can be judged that the polysaccharides contain -CHO and COOH, which confirms the presence of uronic acid. At the same time, it also indicates that there may be a small amount of protein combined with the sugar in the polysaccharides. The absorption peak at 1382 cm^−1^ was caused by the stretching vibrations of C-O in polysaccharides and C-N in proteins. The absorption peak around 1057 cm^−1^ was the common stretching vibration peak of the pyranose ring, including the stretching vibration absorption peaks of C-O-C and C-O-H, indicating that the five PYps were pyranose-ring sugars. An absorption peak was found at 875 cm^−1^, which was a characteristic absorption peak of the C-H vibration of β-terminal isomers, indicating that the five PYps had β-glycosidic bonds [17,18,19]. The absorption at 806 cm^−1^ was the angular vibration of the C-H bond in the D-type furan ring. Combined with the chemical composition analysis, the five PYps may all be complexes of polysaccharides and proteins [18]. These analysis results indicate that the five different extraction methods all yield polysaccharide-like compounds, which all have typical characteristic absorptions of polysaccharides, and the carbon-chain skeletons of the polysaccharides are basically the same. The infrared spectra of the five PYps are shown in Figure 3.

### 2.4. Thermogravimetric Analysis Results 

**Figure 4:**
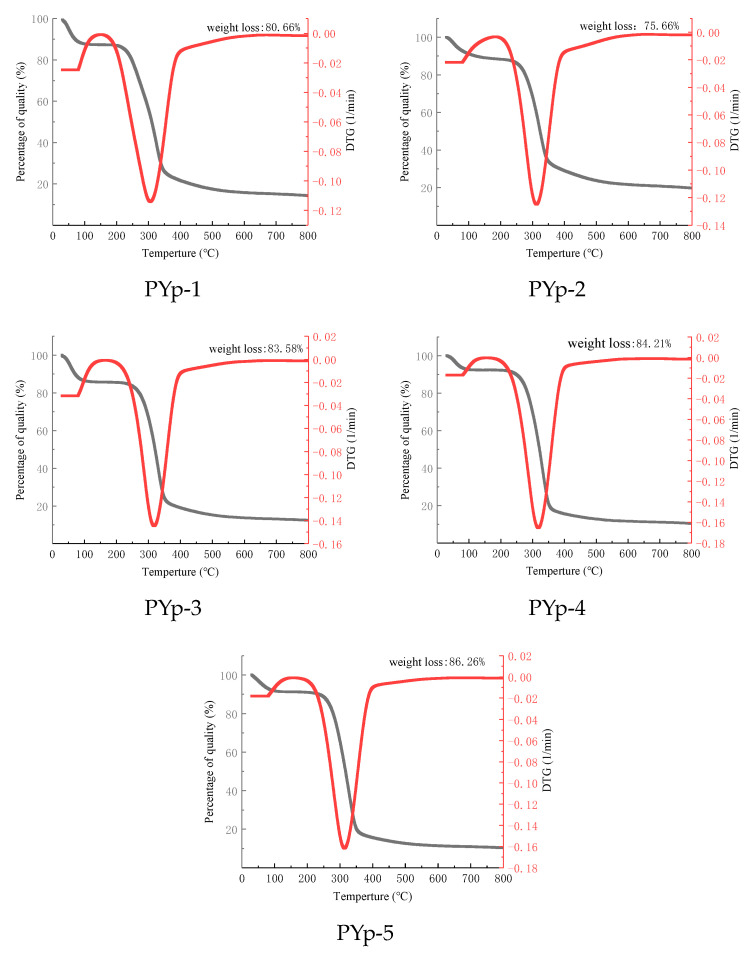
Thermal stability results of five polysaccharides. The black line represents the TG of the PYps, and the red line represents the DTG of the PYps.

During the entire test period, the mass losses of the five PYps followed a similar trend, and their characteristic curves mainly showed a three-step degradation pattern. The first stage occurred at 30–211 °C, which may be related to the evaporation of free water and bound water in the polysaccharide samples. The mass loss rates of PYp1-5 were 12.76%, 11.86%, 14.49%, 7.72%, and 9.06%, respectively, and the mass loss rates were around 10%, indicating that the obtained PYps had a relatively high content of bound water. The second stage occurred at 170–490 °C, where there was an obvious weight loss, mainly due to the loss of crystal water in the polysaccharide samples and the depolymerization of the polysaccharide structure [20]. The mass loss curve became gentle. The mass loss rates of the five PYps were 80.7%, 75.66%, 83.59%, 84.21%, and 84.26%, respectively. The mass loss rate of PYp1-5 at this stage represents the thermal stability of the polysaccharides themselves. At 390–800 °C, the thermal decomposition of the PYp1-5 was basically completed, and the mass gradually became stable. The final remaining mass percentages were 24.34%, 16.42%, 15.79%, 15.74%, and 19.30%, respectively. The thermogravimetric analysis results of PYp1-5 are shown in Figure 4.

### 2.5. Nuclear Magnetic Resonance Hydrogen Spectrum

**Figure 5:**
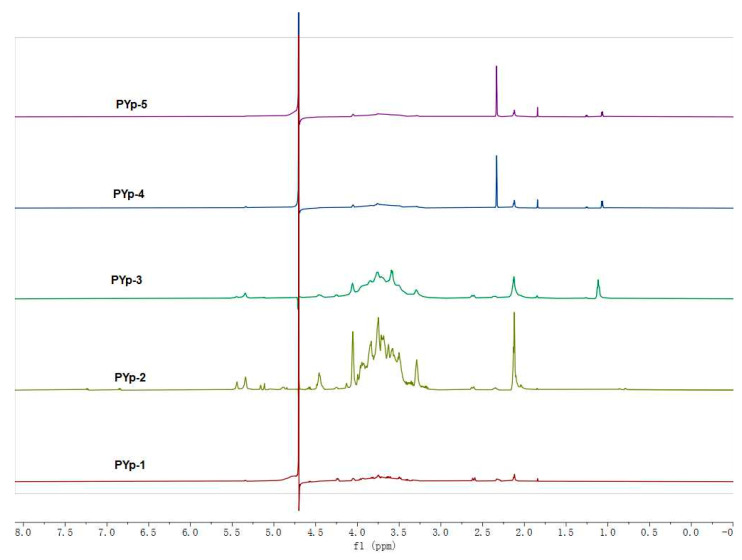
Nuclear magnetic hydrogen spectrum of polysaccharides *P. yunnanensis* by different extraction methods.

Signals in the anomeric proton region appeared at δ 4.4–5.4 for PYp-2 and PYp-3. Among them, the anomeric proton signals of α-configured sugar residues were greater than δ 5.0, and the proton signals of β-configured sugar residues were less than δ 5.0 [19]. A large number of polysaccharide proton peaks appeared at δ 3.0–3.9 for PYp-1, PYp-2, and PYp-3, while there were fewer for PYp-4. This part was a typical polysaccharide signal. The signals in the δ 3.1–4.2 region were attributed to the protons on the glycosidic ring [18,20]. The above results indicate that different extraction methods have a certain impact on the structure of PYp. Cellulose extraction and 80 °C hot water extraction have sugar residues of α and β configurations. The peak at δ 4.7 was caused by the deuterated reagent. In addition, there was a strong signal at δ 3.2–3.5, indicating the presence of more -OCH groups, which is consistent with the results of the infrared spectroscopy analysis. The ^1^H-NMR data of the five PYps are shown in Figure 5.

### 2.6. Scanning Electron Microscope Analysis

**Figure 6:**
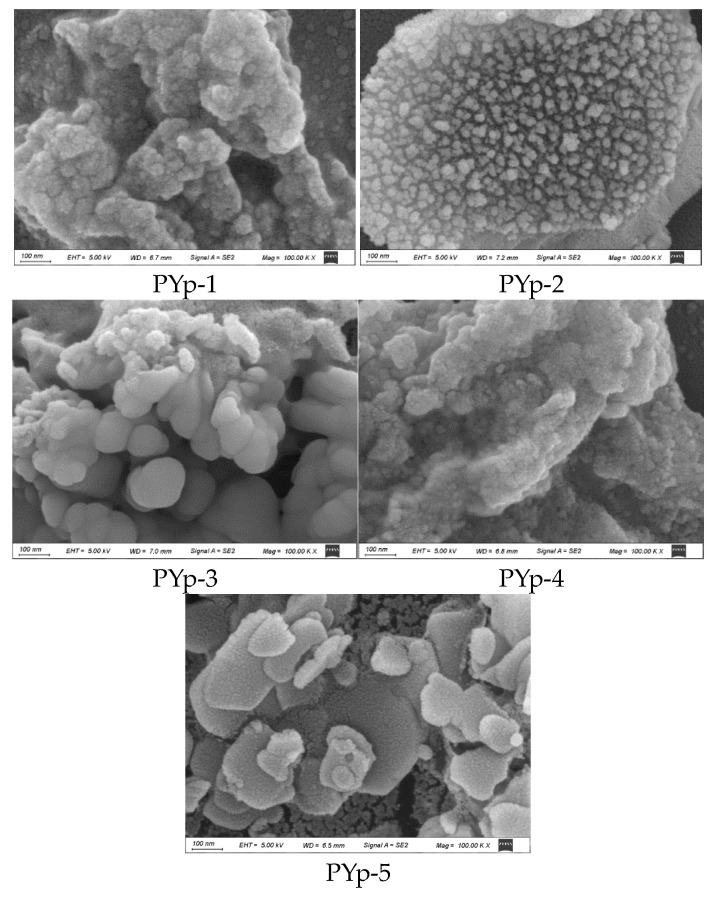
SEM images of *P. yunnanensis* polysaccharides with different extraction methods (100.00 KX× magnifications).

By observing the morphology of the polysaccharides with a scanning electron microscope, their surface characteristics can be analyzed. The apparent morphologies of PYp were mainly irregular blocks, flakes, or granular, with slightly irregular aggregates of debris. PYp-1 and PYp-4 were in the form of dense particles like cotton wool, PYp-2 had granular protrusions and was unevenly distributed. PYp-3 was in the form of smooth-surfaced spheres and was relatively evenly distributed. PYp-5 showed flakes and scattered fine particles. The results indicate that the surface morphologies of the PYp prepared by the different extraction methods were different, not only in shape and particle size but also in surface area. Different treatment methods such as those for PYp-1, PYp-4, and PYp-5 may have caused certain impacts on the polysaccharide structure due to factors such as high heating temperature and long extraction time during the extraction process. The scanning electron microscope images of the five PYps are shown in Figure 6.

### 2.7. In Vitro Antioxidant Activity Analysis

#### 2.7.1. DPPH Radical Scavenging Activity

**Figure 7:**
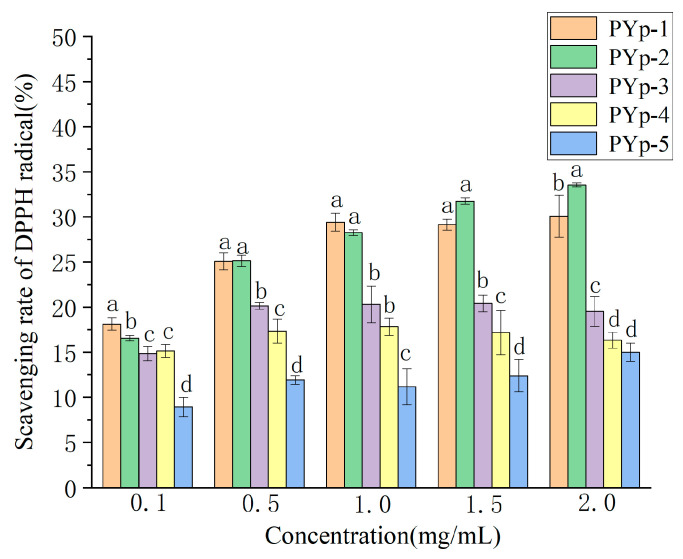
Scavenging ability of *P. yunnanensis* polysaccharides on DPPH radicals. Different letters assigned to each bar indicate significant differences among groups (*p* < 0.05).

As can be seen from Figure 7, at a low concentration of 0.1 mg/mL, the scavenging rates of PYp-1 and PYp-2 were relatively high, while the scavenging rate of PYp-5 was the lowest. As the concentration increased, the scavenging rates of most polysaccharides increased. At the highest concentration of 2.0 mg/mL, the scavenging rates of PYp-1 and PYp-2 were the highest, while the scavenging rate of PYp-5 was still relatively low. The results indicate that the antioxidant activity of PYp-5 was always at the lowest level, while PYp-1 and PYp-2 had good antioxidant activities within the tested concentration range. PYp-1 and PYp-2 may have better antioxidant abilities because their special structures or spatial conformations provide more active groups. The DPPH radical scavenging abilities of the PYps obtained by the different extraction methods are shown in Figure 7.

#### 2.7.2. ABTS^+^ Radical Scavenging Activity

**Figure 8:**
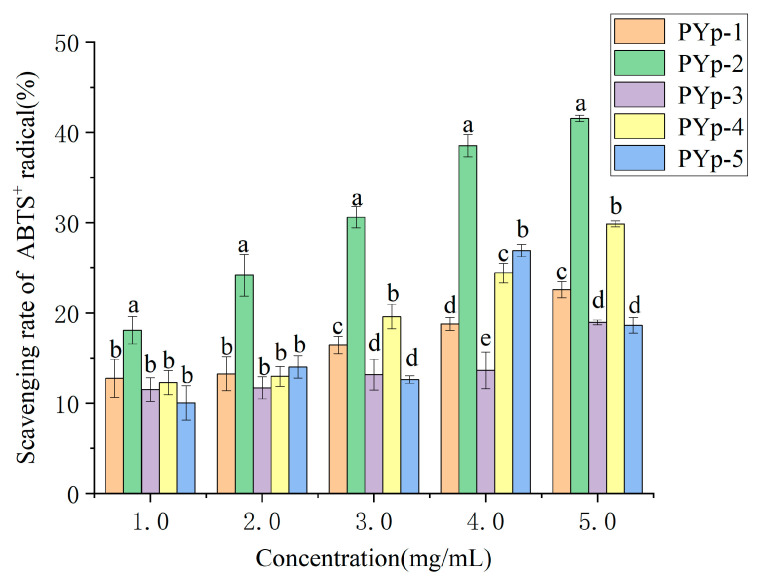
Total antioxidant capacity (ABTs) results. Different letters assigned to each bar indicate significant differences among groups (*p* < 0.05).

The ABTS^+^ radical scavenging abilities of the PYps obtained by the different extraction methods are shown in Figure 8. At a concentration of 1.0 mg/mL, the scavenging rates of PYp-1 and PYp-2 were relatively high, while the scavenging rate of PYp-5 was the lowest. At 5.0 mg/mL, the scavenging rate of PYp-2 was the highest, followed by PYp-4. The scavenging rate of PYp-5 increased, but it was still lower than that of PYp-2. PYp-2 always maintained the highest scavenging rate within the tested concentration range, which may be because PYp-2 had a smaller molecular weight and more exposed active groups. The results indicate that PYp-2 has good potential in ABTS^+^ scavenging.

#### 2.7.3. Superoxide Anion Radical Scavenging Ability

**Figure 9:**
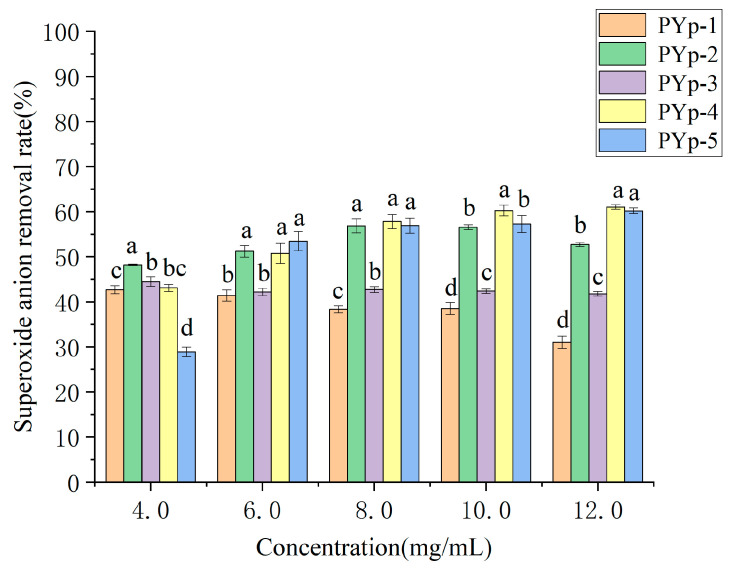
Superoxide anion radical scavenging capacity results. Different letters assigned to each bar indicate significant differences among groups (*p* < 0.05).

The changes in the superoxide anion radical scavenging rates of the PYps obtained by the different extraction methods with their concentrations are shown in Figure 9. The results show that within a certain polysaccharide concentration range, the superoxide anion radical scavenging rate has a certain dose–effect relationship with the polysaccharide concentration. The higher the concentration, the higher the superoxide anion radical scavenging rate. At 4.0 mg/mL, the scavenging rates of PYp-2 and PYp-3 were relatively high, while the scavenging rate of PYp-5 was the lowest. Only the scavenging rates of PYp-1 and PYp-2 decreased after the concentration reached 10.0 mg/mL, but it was not a linear relationship. The scavenging rates of PYp-1 and PYp-3 were always lower than 50%, while the scavenging rates of PYp-2, PYp-4, and PYp-5 were all higher than 50% when the concentration reached 6.0 mg/mL or above. The scavenging rate of PYp-1 was always the lowest starting from a concentration of 6.0 mg/mL, and the scavenging rate of PYp-4 was always the highest starting from a concentration of 8.0 mg/mL. Overall, PYp-4 and PYp-5 have good potential in superoxide anion radical scavenging ability.

### 2.8. In Vitro Hypolipidemic Activity Results

The level of blood lipids is influenced by the cholesterol content in the human body. Cholesterol is converted into bile acids in the liver, which undergo enterohepatic circulation: after lipid digestion, the majority of bile acids are reabsorbed in the small intestine and returned to the liver, while a minor fraction is excreted in feces. Blocking the enterohepatic circulation of bile acids to reduce their reabsorption can increase the excretion of bile acid derivatives, thereby promoting the conversion of more cholesterol into bile acids and achieving the goal of lowering blood cholesterol levels.

In the liver, bile acids mainly exist in the form of conjugates, primarily binding with glycine and taurine to form sodium glycocholate or sodium taurocholate. Therefore, the hypolipidemic potential of samples can be characterized by measuring their binding capacity to bile salts [21].

#### 2.8.1. Sodium Glycocholate Binding Capacity Assay

**Figure 10:**
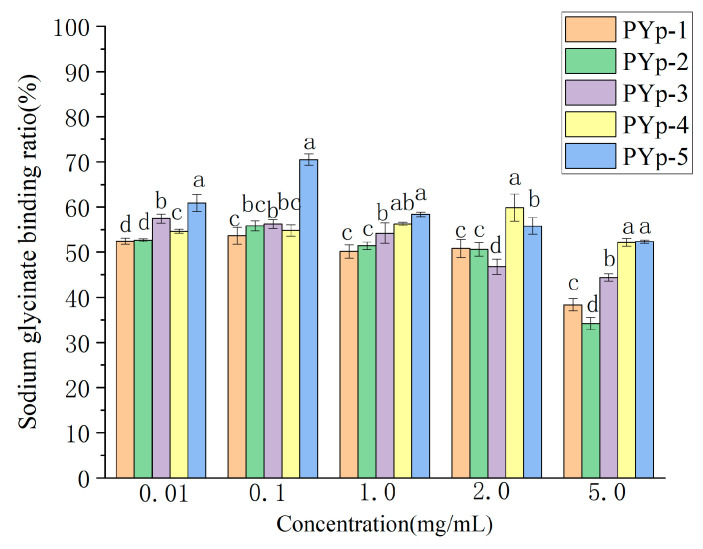
Sodium glycinate binding ability results. Different letters assigned to each bar indicate significant differences among groups (*p* < 0.05).

As can be seen from Figure 10, the PYps obtained by the different extraction methods all had a certain binding ability to sodium glycocholate. In the test range of 0.1–5.0 mg/mL, the binding rates of PYp-1, PYp-2, PYp-3, and PYp-5 all decreased to varying degrees. The binding rates of PYp-2 and PYp-5 to sodium glycinate showed a trend of first increasing and then decreasing in the test range. The performances of PYp-2 and PYp-3 at different concentrations were relatively stable, but the binding rates were generally lower than those of PYp-4 and PYp-5. PYp-4 and PYp-5 showed high binding abilities at all concentrations, which may be related to the structural characteristics of the polysaccharides, such as molecular size, the higher-order conformation of the polysaccharide chain, and charge distribution [22,23,24].

#### 2.8.2. Sodium Taurocholate Binding Capacity Assay

**Figure 11:**
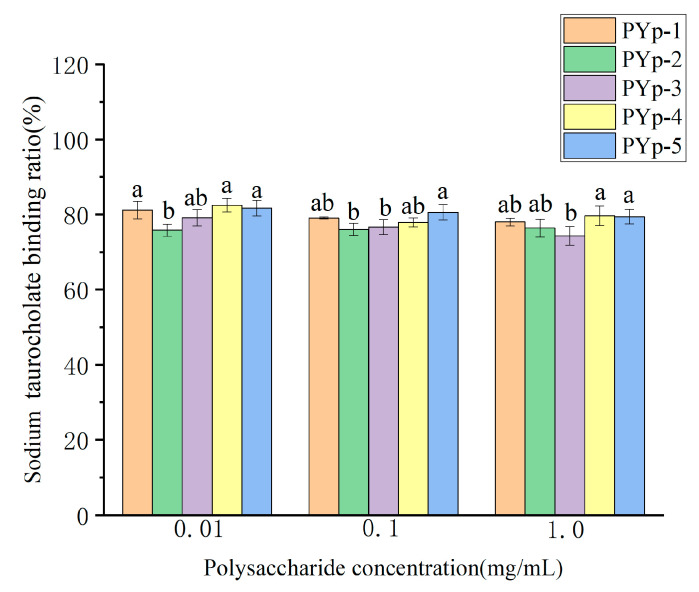
Sodium taurocholate binding capacity results. Different letters assigned to each bar indicate significant differences among groups (*p* < 0.05).

As can be seen from Figure 11, regardless of the extraction method, the PYps could achieve a binding ability to sodium taurocholate of more than 50% under low-concentration conditions. There was no significant difference in PYp-2 and PYp-5 at each concentration, and PYp-5 showed a high binding ratio at all concentrations. Although the binding ratios of PYp-1 and PYp-3 decreased to varying degrees, they remained relatively stable. The binding ratio of PYp-2 was relatively stable with no obvious change. From the figure, it can be seen that regardless of the extraction method, the polysaccharides from *P. yunnanensis* could maintain a good binding rate with sodium taurocholate under low-concentration conditions. The treatments of PYp-1, PYp-4, and PYp-5 performed better. This may be because ultrasonic and microwave treatments can more effectively destroy the crystal structure of the polysaccharides and increase their surface area, thereby improving the binding ability. In contrast, the binding ratio of PYp-2 was relatively low, which may be because the enzyme treatment may have changed the molecular structure of the polysaccharides and affected their binding ability to sodium taurocholate. This result is of great significance for selecting suitable polysaccharide treatment methods to improve their biological activities.

## 3. Materials and Methods

### 3.1. Chemical Reagents

Nitrogen purging instrument (Reacti-thermo, Thermo Fisher Scientific, Waltham, MA, USA) and Fourier Transform Infrared Spectrometer (Nicolet 20, Nicolet Scientific, Waltham, MA, USA): Thermo Fisher Scientific (Waltham, MA, USA); Vortex mixer (XH-T): Xinbao Instrument (Changzhou, China); ion chromatography (ICS 5000+): Thermo Fisher Scientific (Waltham, MA, USA); scanning electron microscope (Zeiss Supra 55): Carl Zeiss AG (Oberkochen, Germany); Freeze-dryer (CTFD-185–80 °C): Qingdao Yonghe Chuangxin Electronic Technology Co., Ltd. (Qingdao, China); microplate reader (INFINITE 200 PRO): Tecan Austria GmbH (Salzburg, Austria); Electronic balance (BSA224S-CW): Sartorius Scientific Instruments (Beijing) Co., Ltd. (Beijing, China); Ultrasonic Cleaning Machine (SG8200HBT): Shanghai Guante Ultrasonic Instrument Co., Ltd. (Shanghai, China). Microwave oven (G80F20CN2L-B8 (RO)): Galanz Group (Zhongshan, China); UV-Vis spectrophotometer (UV-1800PC): Aoyi Instruments Co., Ltd. (Shanghai, China); Nuclear Magnetic Resonance (NMR) Spectrometer (BRUKER AVANCEIII HD500): Bruker BioSpin GmbH (Ettlingen, Germany);

Chemical Reagents: Sodium glycocholate, sodium taurocholate, 1,1-diphenyl-2-picrylhydrazyl (DPPH), bovine serum albumin, trypsin (S10032, 250 µ/mg), pepsin (S10027, 1:30,000)—Shanghai Yuanye Biotechnology Co., Ltd. (Shanghai, China); superoxide anion determination kit—Suzhou Keming Biotechnology Co., Ltd. (Suzhou, China); Trifluoroacetic acid (76-05-1, chromatographic grade, ANPEL), sodium hydroxide (1310-73-2, chromatographic grade)—Sigma-Aldrich (St. Louis, MO, USA); Fucose (2438-80-4), Rhamnose (10030-85-0), Arabinose (5328-37-0), Galactose (26566-61-0), glucose (50-99-7), Xylose (58-86-6), mannose (3458-28-4), Fructose (57-48-7), Ribose (50-69-1), galacturonic acid (14982-50-4), glucuronic acid (6556-12-3), mannuronic acid (6814-36-4), guluronic acid (15769-56-9)—all of the above reagents were purchased from Sigma (St. Louis, MO, USA). Note: All the reagents are of analytical grade.

### 3.2. Extraction of P. yunnanensis Rolfe Polysaccharides

#### 3.2.1. Preparation of Sample

Two thousand grams of *P. yunnanensis* pseudobulbs was collected, dried, and ground into powder. The powder was then passed through a No. 5 mesh, followed by degreasing with petroleum ether at a solid–liquid ratio of 1:5 for 24 h. The degreasing process was repeated twice. After complete solvent volatilization, the degreased powder was stored for further use [14].

#### 3.2.2. Extraction Protocols

##### Microwave-Assisted Extraction

General Procedure (Common to All Methods):

Initial Preparation: A total of 5.0 g of degreased powder was homogenized with distilled water (1:20, *w*/*v*).

Microwave-assisted (PYp-1): Extraction was conducted at 800 W for 10 min using a microwave oven (G80F20CN2L - B8 (RO), Galanz Group, Zhongshan, China)..

Enzyme-assisted (PYp-2): It included 1% (*w*/*w*) cellulase, pH 5.0, 45 °C/1 h with intermittent stirring twice, followed by enzyme inactivation (90 °C/10 min).

Hot water (PYp-3): An 80 °C water bath for 1.5 h with stirring twice.

Ultrasound-assisted (PYp-4): A 500 W, 59 kHz (SG8200HBT, Shanghai Guante Ultrasonic Instrument Co., Ltd. Shanghai, China) sonication (room temperature) for 60 min.

Cold water (PYp-5): Immerse it at 4 °C for 12 h, and manually stir it twice during this period.

Post-Extraction Processing:

Each of the five extraction methods was performed twice. The filtrates were combined and centrifuged at 5000 rpm/min for 20 min. The combined supernatants were concentrated to 50 mL, mixed with ethanol at a ratio of 1:4 (*v*/*v*) at 4 °C overnight, filtered, and centrifuged. The precipitate was washed with 95% ethanol and then lyophilized to obtain the PYp-1–5.

### 3.3. Physicochemical Characterization

#### 3.3.1. Total Polysaccharide Content Determination

A standard curve for polysaccharides was established using the phenol–sulfuric acid method with anhydrous glucose as the reference standard according to the established methodology.

A linear regression analysis correlating glucose concentration (x-axis, mg/mL) with absorbance (y-axis) (microplate reader, INFINITE 200 PRO, Tecan Austria GmbH, Salzburg, Austria) produced the standard curve:

y = 6.5924x + 0.3887, R^2^ = 0.9994.

For sample analysis, 25.00 mg of each polysaccharide was dissolved in distilled water and volumetrically adjusted to 50 mL. Aliquots (0.4 mL) of the sample solutions were subjected to the same analytical procedure. The total polysaccharide content was calculated based on the derived standard curve.

#### 3.3.2. Uronic Acid Content Determination

The uronic acid content was determined using the sulfuric acid–borax–carbazole method with glucuronic acid as the standard [17]. A stock solution of glucuronic acid (20.00 mg) was accurately weighed, dissolved in distilled water, and diluted to 100 mL. The solution was further diluted to generate standard concentrations of 0.02, 0.06, 0.10, 0.12, 0.16, and 0.20 mg/mL. For the assay, 0.1 mL of each standard solution was mixed with 0.6 mL of sulfuric acid–borax solution and 0.2 mL of carbazole solution. The mixtures were heated at 100 °C for 10 min, cooled to room temperature, and measured (microplate reader, INFINITE 200 PRO, Tecan Austria GmbH, Salzburg, Austria) for absorbance at 530 nm.

The standard curve exhibited the following linear relationship:

y = 3.4981x + 0.0825, R^2^ = 0.9957.

where x represents the glucuronic acid concentration (mg/mL).

For sample analysis, 10.00 mg of each PYP was dissolved in distilled water and diluted to 100 mL. Aliquots (0.1 mL) of the solutions were processed identically, and the uronic acid content was calculated using the regression equation.

#### 3.3.3. Protein Content Determination

The protein content was analyzed using the Bradford method with bovine serum albumin (BSA) as the reference standard [20]. A stock solution of BSA (5.00 mg) was accurately weighed, dissolved in physiological saline, and diluted to 10 mL. Serial dilutions were prepared to achieve standard concentrations of 0.0, 20, 40, 60, 80, 100, and 120 μg/mL. For quantification, 1 mL of Coomassie Brilliant Blue solution was added to 200 μL of each standard solution. After incubation in darkness for 10 min, absorbance was measured (UV-Vis spectrophotometer UV-1800PC, Aoyi Instruments Co., Ltd. Shanghai, China) at 595 nm.

The standard curve demonstrated the linear relationship:

y = 0.0079x + 0.6265, R^2^ = 0.9912.

where x corresponds to the BSA concentration (μg/mL). For the polysaccharide samples, 10.00 mg of each PYP was dissolved in distilled water and diluted to 10 mL. Aliquots (200 μL) were analyzed following the same protocol, and the protein content was determined using the standard curve.

#### 3.3.4. Monosaccharide Composition Analysis

We weighed 20 mg of each of the five extracted polysaccharides, added 1 mL of 2 M Trifluoroacetic acid (TFA) solution, mixed well, and heated at 121 °C for 2 h. We purged them with nitrogen and dried them. We added methanol for washing and then dried again. We repeated the methanol washing 2–3 times. We added 1 mL of sterile water to dissolve and transfer them into a chromatographic vial for testing. The monosaccharide components were analyzed and detected using a Thermo ICS 5000+ ion chromatography system (ICS 5000+, Thermo Fisher Scientific, USA) equipped with an electrochemical detector. A Dionex™ CarboPac™ PA20 liquid chromatography column (Thermo Fisher Scientific, Waltham, MA, USA, 150 × 3.0 mm, 10 μm) was employed for separation, with an injection volume of 5 μL. The analysis was performed at a flow rate of 0.5 mL/min under isocratic elution conditions. The column temperature was maintained at 30 °C, mobile phase A (H_2_O), mobile phase B (0.1 M NaOH), mobile phase C (0.1 M NaOH, 0.2 M NaAc), flow rate 0.5 mL/min; the column temperature was 30 °C; the elution program is shown in Table 2 [25,26,27].

#### 3.3.5. Molecular Weight Determination

The molecular weight of the polysaccharides was determined by gel permeation chromatography (GPC) (PL-GPC50, Agilent Technologies Inc. UK). The five PYp samples were prepared into 5 mg/mL aqueous solutions, respectively, and filtered with a 0.45 μm filter membrane. The GPC analysis was carried out using an Agilent GPC 50 gel permeation chromatography system, with a PL mix—c column (7.5 × 50 mm, 5 μm) and a PL mix—c column (7.5 × 300 mm, 5 μm) in series. The column temperature was maintained at 25 °C using dimethyl sulfoxide as the mobile phase with a flow rate of 1 mL/min. Data were collected using a differential refractive index detector. Abbreviations: Mw—weight average molecular weight; Mn—number average molecular weight; PDI—polydispersity index. The percentage values in parentheses represent the standard error estimation calculated by the MALLS detector.

#### 3.3.6. Fourier Transform Infrared (FTIR) Analysis

Five PYp samples were ground into powder and passed through a 60-mesh sieve. Then, 20 mg of each sample was accurately weighed and tableted using the KBr pellet method for detection. The scanning range (Fourier Transform Infrared Spectrometer, Nicolet 20, formerly Nicolet Scientific, Waltham, MA, USA)was set from 400 to 4000 cm^−1^ [28].

#### 3.3.7. Nuclear Magnetic Resonance Hydrogen Spectrum Analysis

We accurately weighed 20 mg of each of the five PYp samples, dissolved them in 0.5 mL of D_2_O, and performed ^1^H-NMR testing (Nuclear Magnetic Resonance (NMR) Spectrometer, BRUKER AVANCEIII HD500, Bruker BioSpin GmbH, Ettlingen, Germany) under a 500 M magnetic field [15].

#### 3.3.8. Scanning Electron Microscope (SEM) Analysis

We took 10 mg of the PYp sample and adhered it to the sample stage. We placed it in a vacuum sputtering coater to coat a conductive layer. The accelerating voltage range was 0.1 KV–30 kV, and the landing energy range was 20 eV–30 keV. We observed with a Schottky thermal field emission scanning electron microscope (Zeiss Supra 55, Carl Zeiss AG, Oberkochen, Germany) to analyze the surface structural morphology of the different samples.

#### 3.3.9. Thermogravimetric Analysis

We weighed precisely 200 mg of each of the five PYp samples and carefully placed them onto the sample pans. The thermogravimetric analysis was then performed using a Mettler-Toledo TGA2 thermogravimetric analyzer under a nitrogen atmosphere. For the measurement, the heating rate was set at 20 °C/min, and the temperature range was defined from 30 °C to 800 °C. This allowed for comprehensive thermogravimetric analysis (TG/DTG) (Switzerland Mettler TGA2, Mettler-Toledo, Greifensee, Switzerland) of the samples.

### 3.4. Assay for Antioxidant Activity

#### 3.4.1. DPPH Radical Scavenging Ability Determination

We prepared the five polysaccharides into sample test solutions with concentrations of 0.1, 0.5, 1.0, 1.5, and 2.0 mg/mL, respectively. We took each test solution and mixed it with the DPPH solution (prepared with ethanol, 0.2 mmol/L) at a volume ratio of 1:1 and added them to a 96-well microplate. After mixing well, they reacted at room temperature in the dark for 30 min. We used distilled water as a blank control and measured (microplate reader, INFINITE 200 PRO, Tecan Austria GmbH, Salzburg, Austria) the absorbance at a wavelength of 517 nm [29]. Each test was performed in triplicate. The DPPH radical scavenging rate was calculated (clearance, CL) using Formula (1):

(1) $${\mathrm{CL}}_{\mathrm{DPPH}}=1-\frac{A_{1}-A_{2}}{A_{0}}\times 100\%$$

where A_0_: absorbance of the blank group sample;

A_1_: absorbance of the sample group;

A_2_: absorbance of the control group sample.

#### 3.4.2. ABTS^+^ Radical Scavenging Ability Determination

We prepared the five polysaccharides into sample test solutions with concentrations of 1.0, 2.0, 3.0, 4.0, and 5.0 mg/mL, respectively. We took each test solution and mixed it with the ABTS reaction solution (ABTS and K_2_S_2_O_8_) at a volume ratio of 1:1 and added them to a 96-well microplate. They were shaken well and reacted at room temperature in the dark for 5 min. We used distilled water as a blank control and measured (microplate reader, INFINITE 200 PRO, Tecan Austria GmbH, Salzburg, Austria) the absorbance at 734 nm [30]. Each test was performed in triplicate. The ABTS radical scavenging rate was calculated according to Formula (2).

(2) $${{\mathrm{CL}}_{\mathrm{ABTS}}}^{+}=1-\frac{A_{1}-A_{2}}{A_{0}}\times 100\%$$

where A_0_ is the absorbance value measured when distilled water replaces the sample solution;

A_1_ is the absorbance value of the PYp solution;

A_2_ is the absorbance value when an equal volume of phosphate buffer replaces the ABTS radical working solution.

#### 3.4.3. Superoxide Anion Radical Scavenging Ability Determination

We prepared the five polysaccharides into test solutions with concentrations of 4.0, 6.0, 8.0, 10.0, and 12.0 mg/mL, respectively. We added 10 μL of reagent 1 and 40 μL of reagent 2 to the test tubes in sequence, mixed well, and reacted at 25 °C for 1 min. Then, add 25 μL of PYp and 50 μL of reagent 3, respectively, were mixed thoroughly and reacted at 37 °C for 30 min. Finally, we added reagent 4 and reagent 5, mixed well, and developed color at 37 °C for 20 min. We used distilled water as a blank control and measured (microplate reader, INFINITE 200 PRO, Tecan Austria GmbH, Salzburg, Austria) the absorbance at 530 nm. Each test was performed in triplicate. The superoxide anion scavenging rate was calculated according to Formula (3).

(3) $$I\%=\frac{A_{0}-A_{1}}{A_{0}}\times 100\%$$

where I is the superoxide anion radical scavenging rate;

A_0_ is the absorbance value of the control tube;

A_1_ is the absorbance value of the test tube.

### 3.5. In Vitro Hypolipidemic Ability Determination

#### 3.5.1. Determination of Sodium Glycocholate Binding Ability

We accurately measured 10 mg of sodium glycocholate and prepared it into different concentration gradients. We took 0.5 mL of solutions with different concentrations, added 1.5 mL of 60% concentrated H_2_SO_4_, shook them well, and placed them in a 70 °C constant-temperature water bath for 20 min. We took them out and ice-bathed them for 5 min. We measured (microplate reader, INFINITE 200 PRO, Tecan Austria GmbH, Salzburg, Austria) the absorbance value at 387 nm using a microplate reader. A standard calibration curve was generated by plotting absorbance values (y-axis) against sodium glycocholate concentrations (x-axis) using linear regression analysis.

We prepared the PYps into a 10 mg/mL solution, and then prepared test solutions with different concentrations. We precisely pipetted 0.3 mL of each into a 2 mL test tube, added 0.1 mL of pepsin (prepared to a concentration of 10 mg/mL with 0.1 mol/L phosphate buffer at pH 6.3), 0.3 mL of 0.01 mol/L hydrochloric acid solution, and shook at a constant temperature of 37 °C for 1 h to simulate gastric digestion. Then, we added 0.3 mL of 0.1 mol/L sodium hydroxide solution, followed by 0.4 mL of trypsin (prepared to a concentration of 10 mg/mL with 0.1 mol/L phosphate buffer at pH 6.3) and 0.4 mL of sodium glycocholate solution (prepared to a concentration of 2 mg/mL with 0.1 mol/L phosphate buffer at pH 6.3), and shook at a constant temperature of 37 °C for 1 h to simulate small-intestine digestion. Then, we transferred the mixture to a centrifuge tube, centrifuged at 8000 r/min for 10 min, took 0.5 mL of the supernatant, added 1.5 mL of 60% concentrated H_2_SO_4_, shook well, placed it in a 70 °C constant-temperature water bath for 20 min, took it out and ice-bathed it for 5 min, and measured the absorbance value at 387 nm using a microplate reader [29]. The sodium glycocholate binding rate was calculated using Formula (4):

(4) $$\mathrm{Sodium}\;\mathrm{glycocholate}\;\mathrm{binding}\;\mathrm{rate}=\frac{amount\;of\;added\;sodium\;glycinate-remaining\;amount}{amount\;of\;added\;sodium\;glycinate}\times 100\%$$

#### 3.5.2. Determination of Sodium Taurocholate Binding Ability

We accurately measured 10 mg of sodium taurocholate and prepared it into different concentration gradients. We took 0.5 mL of sodium taurocholate solutions with different concentrations, added 1.5 mL of 60% concentrated H_2_SO_4_, shook them well, placed them in a 70 °C constant-temperature water bath for 20 min, took them out, and ice-bathed them for 5 min. We measured (microplate reader, INFINITE 200 PRO, Tecan Austria GmbH, Salzburg, Austria) the absorbance value at 387 nm using a microplate reader. A calibration curve was constructed by plotting absorbance values (y-axis) against sodium taurocholate concentrations (x-axis) using linear regression analysis [29].

We prepared the five polysaccharides into a 10 mg/mL solution, respectively, and then diluted them to concentrations of 0.01, 0.1, and 1.0 mg/mL. We precisely pipetted 0.3 mL of each test solution into a 2 mL test tube, added 0.1 mL of 10 mg/mL pepsin (prepared to a concentration of 10 mg/mL with 0.1 mol/L phosphate buffer at pH 6.3), 0.3 mL of 0.01 mol/L hydrochloric acid solution, and shook at a constant temperature of 37 °C for 1 h to simulate gastric digestion. Then, we added 0.3 mL of 0.1 mol/L sodium hydroxide solution, followed by 0.4 mL of trypsin (prepared to a concentration of 10 mg/mL with 0.1 mol/L phosphate buffer at pH 6.3) and 0.4 mL of sodium taurocholate (prepared to a concentration of 2 mg/mL with 0.1 mol/L phosphate buffer at pH 6.3), and shook at a constant temperature of 37 °C for 1 h to simulate small-intestine digestion. Then, we transferred the mixture to a centrifuge tube, centrifuged at 8000 r/min for 10 min, took 0.5 mL of the supernatant, added 1.5 mL of 60% concentrated H_2_SO_4_, shook well, placed it in a 70 °C constant-temperature water bath for 20 min, took it out and ice-bathed it for 5 min, and measured the absorbance value at 387 nm using a microplate reader. The sodium taurocholate binding rate was calculated using Formula (5):

(5) $$\mathrm{Sodium}\;\mathrm{taurocholate}\;\mathrm{binding}\;\mathrm{rate}=\frac{amount\;of\;added\;sodium\;taurocholate-remaining\;amount}{amount\;of\;added\;sodium\;taurocholate}$$

### 3.6. Statistical Analysis

All the experimental results were repeated at least three times and expressed as mean ± standard deviation. ANOVA and Duncan’s test (*p* < 0.05) were used to analyze the significant differences. The SPSS 25.0 software was used for the correlation analysis, and the Origin 2019 software was used for graphic processing.

## 4. Conclusions

It was found that the enzymatic extraction method had the advantages of low energy consumption, environmental friendliness, and energy conservation. The polysaccharide obtained by this method had the best antioxidant activity. This might be because the enzymatic extraction process was relatively mild and had a certain decomposition effect on the polysaccharide, resulting in a reduced molecular weight and thus, good antioxidant activity. This also implies that low-molecular-weight polysaccharides have better antioxidant activity. In the in vitro hypolipidemic experiment, the polysaccharides extracted by ultrasonic and cold water methods had better effects. Considering the actual production, ultrasonic extraction had the characteristics of low cost, short time, and high efficiency, making it a suitable extraction method for developing adjuvant hypolipidemic products. However, the antioxidant and hypolipidemic active substances of *P. yunnanensis* polysaccharides might have thermal instability, which still needs further in-depth research and verification.

This study systematically evaluated the polysaccharides obtained by the different extraction methods from multiple levels and angles of structure—physicochemistry—efficacy, integrating the knowledge of analytical chemistry, pharmacodynamics, and other disciplines, providing a theoretical basis for the development and utilization of polysaccharides. However, in this experiment, the polysaccharides were not isolated and purified, and in vivo antioxidant and hypolipidemic studies were not carried out. The binding targets of polysaccharides and the pathway mechanisms and physiological mechanisms of their actions were not explored, so the impact of extraction methods on the structure-function relationship of polysaccharides was not fully revealed. In the future, relevant research needs to be further carried out to deeply analyze the relationship between the fine structure and function of polysaccharides, providing a reference for the research and development of functional and health-care foods and adjuvant drugs.

## Figures and Tables

**Table 1:** Effect of different extraction methods on monosaccharide composition of polysaccharides from *Pleione yunnanesis* (*n* = 3).

Standard	Proportion/%				
Product	PYp-1	PYp-2	PYp-3	PYp-4	PYp-5
Fuc	-	-	-	-	-
Ara	-	-	-	-	-
Rha	-	-	-	-	-
Gal	-	-	-	-	-
Glc	45.81 ± 0.025	50.37 ± 0.040	44.89 ± 0.080	44.61 ± 0.012	43.85 ± 0.015
Xyl	-	-	-	-	-
Man	54.19 ± 0.010	49.63 ± 0.006	55.11 ± 0.015	55.39 ± 0.025	56.15 ± 0.010
Fru	-	-	-	-	-
Rib	-	-	-	-	-
Gal-UA	-	-	-	-	-
Gul-UA	-	-	-	-	-
Glc-UA	-	-	-	-	-
Man-UA	-	-	-	-	-

Note: “-” indicates not detected.

**Table 2:** Elution program.

Time (min)	Phase A (H_2_O) %	B-Phase (0.1 M NaOH) %	(C Phase 0.1 M NaOH, 0.2 M NaAc) %
0	95	5	0
26	85	5	10
42	85	5	10
42.1	60	0	40
52	60	40	0
52.1	95	5	0
60	95	5	0

## Data Availability

The datasets used or analyzed during the current study are available from the corresponding author upon reasonable request.
